# Supplementary material for: More is not enough: High quantity and high quality antenatal care are both needed to prevent low birthweight in South Asia
Source: PLOS Glob Public Health. 2023 Jun 8;3(6):e0001991. doi: 10.1371/journal.pgph.0001991 (PMC10249805; doi:10.1371/journal.pgph.0001991)
Supplement: S1 Fig — Shape file accessible at: https://gadm.org/download_country_v3.html. (DOCX) [file pgph.0001991.s001.docx]

| 1. **Afghanistan, 2015** | 1. **Bangladesh, 2018** |
| --- | --- |
| 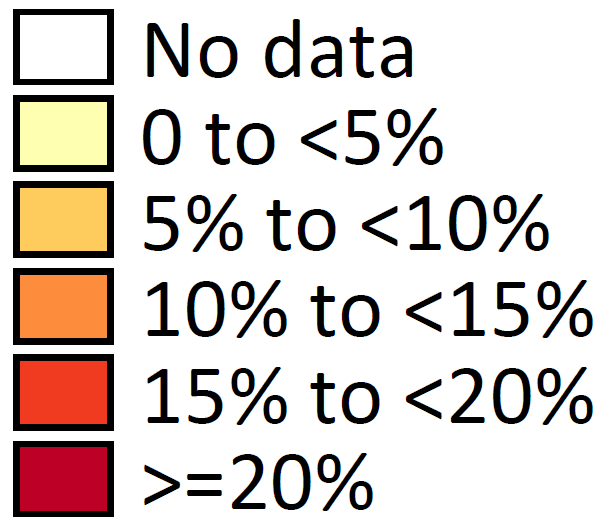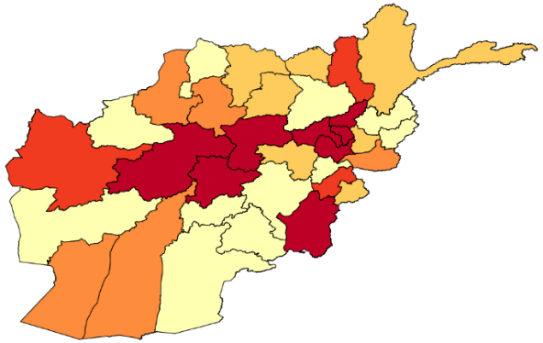 | 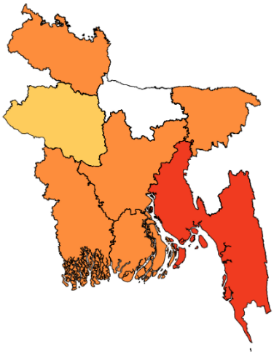 |
|  |  |
| 1. **India, 2016** | 1. **Nepal, 2016** |
| **** | 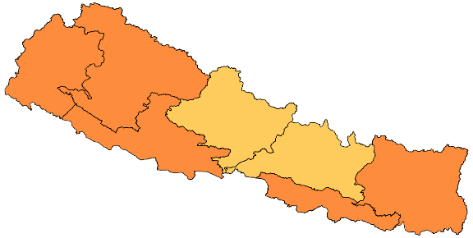 |
| 1. **Pakistan, 2018** | 1. **Sri Lanka, 2016** |
| 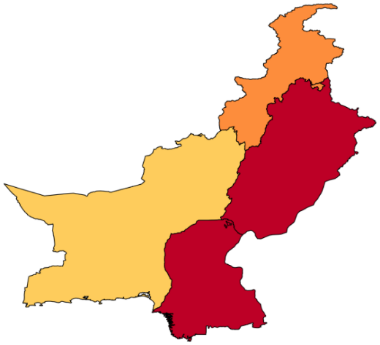 | 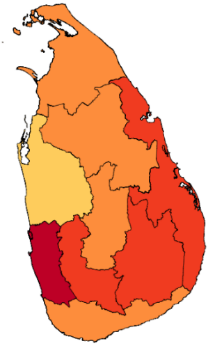 |
|  | |

Shape file accessible at: https://gadm.org/download_country_v3.html
