# Supplementary material for: More is not enough: High quantity and high quality antenatal care are both needed to prevent low birthweight in South Asia
Source: PLOS Glob Public Health. 2023 Jun 8;3(6):e0001991. doi: 10.1371/journal.pgph.0001991 (PMC10249805; doi:10.1371/journal.pgph.0001991)
Supplement: S1 Table — (DOCX) [file pgph.0001991.s002.docx]

|  | Afghanistan  2015 | | Bangladesh  2018^1^ | India  2016 | | Nepal  2016 | | Pakistan  2018 | | Sri Lanka  2016 |
| --- | --- | --- | --- | --- | --- | --- | --- | --- | --- | --- |
|  | Birth weight | Birth size^1^ | Birth  weight | Birth  weight | Birth  size | Birth  weight | Birth size | Birth weight | Birth size | Birth  weight |
|  |  |  |  |  |  |  |  |  |  |  |
| No. youngest children under five years of age | 19,689 | 19,689 | 5,012 | 190,898 | 190,898 | 4,006 | 4,006 | 8,286 | 8,286 | 7,040 |
| *Excluded due to missing data on birth weight or perceived birth size* | *→16,906* | *→504* | *→2,733* | *→43,136* | *→6,971* | *→1,388* | *→64* | *→6,665* | *→1,656* | *→130* |
| Analytical sample  (Recorded + Recalled birthweight | 2,783 | 19,185 | 2,279 | 147,674 | 183,927 | 2,618 | 3,942 | 1,621 | 6,630 | 6,910 |
| Recorded birthweight | 1,312 | - | 95 | 80,504 | - | 521 | - | 214 | - | 6,910 |
| Recalled birthweight | 1,471 | - | 2,184 | 67,170 | - | 2,097 | - | 1,407 | - | - |
| ^1^Birth size is a perceived size of a child by the mother (very small, small, normal, large, and very large), data on perceived size were not available for Bangladesh and Sri Lanka. | | | | | | | | | | |
