# Supplementary material for: More is not enough: High quantity and high quality antenatal care are both needed to prevent low birthweight in South Asia
Source: PLOS Glob Public Health. 2023 Jun 8;3(6):e0001991. doi: 10.1371/journal.pgph.0001991 (PMC10249805; doi:10.1371/journal.pgph.0001991)
Supplement: S2 Table — (DOCX) [file pgph.0001991.s003.docx]

| **AFGHANISTAN, 2015** |  | BANGLADESH, 2018 (cont.) |  | INDIA, 2016 (cont.) |  |
| --- | --- | --- | --- | --- | --- |
| Badakhshan | 10.9 | Khulna | 15.3 | Tripura | 13.9 |
| Badghis | 2.9 | Rajshahi | 9.7 | Uttar Pradesh | 21.3 |
| Baghlan | 4.2 | Rangpur | 15.8 | Uttaranchal | 8.3 |
| Balkh | 5.1 | Sylhet | 13.4 | West Bengal | 17.0 |
| Bamyan | 58.5 | Barisal | 18.8 | **NEPAL, 2016** |  |
| Daykundi | 33.2 | **INDIA, 2016** |  | Province 1 | 12.5 |
| Farah | 2.1 | A & N Islands | 16.0 | Province 2 | 12.0 |
| Faryab | 13.2 | Andhra Pradesh | 19.7 | Province 3 | 8.7 |
| Ghazni | 3.6 | Arunachal Pradesh | 17.8 | Province 4 | 8.7 |
| Ghor | 62.3 | Assam | 19.9 | Province 5 | 13.5 |
| Helmand | 11.3 | Bihar | 14.8 | Province 6 | 12.1 |
| Herat | 17.4 | Chandigarh | 11.8 | Province 7 | 11.7 |
| Jawzjan | 2.6 | Chattisgarh | 20.1 | **PAKISTAN, 2018** |  |
| Kabul | 21.1 | D & N Haveli | 23.8 | Balochistan | 9.8 |
| Kandahar | 2.7 | Daman & Diu | 8.6 | Fata | 6.3 |
| Kapisa | 21.8 | Delhi | 8.1 | Islamabad (ICT) | 15.9 |
| Khost | 8.8 | Goa | 21.4 | Khyber Pakhtunkhwa | 14.4 |
| Kunarha | 3.7 | Gujarat | 16.9 | Punjab | 25.2 |
| Kunduz | 8.7 | Haryana | 15.1 | Sindh | 22.0 |
| Laghman | 5.1 | Himachal Pradesh | 18.6 | **SRI** **LANKA, 2016** |  |
| Logar | 3.1 | Jammu & Kashmir | 20.8 | Central province | 16.2 |
| Nangarhar | 12.0 | Jharkhand | 17.1 | Eastern province | 15.8 |
| Nimroz | 12.5 | Karnataka | 15.6 | North central province | 14.6 |
| Nooristan | 0.0 | Kerala | 5.2 | North western province | 16.2 |
| Paktika | 25.3 | Lakshadweep | 16.5 | Northern province | 9.5 |
| Paktya | 19.0 | Madhya Pradesh | 13.6 | Sabaragamuwa province | 21.3 |
| Panjsher | 25.2 | Maharastra | 14.4 | Southern province | 14.0 |
| Parwan | 31.9 | Manipur | 13.8 | Uva province | 16.3 |
| Samangan | 5.5 | Meghalaya | 19.3 | Western province | 13.7 |
| Sar-E-Pul | 13.3 | Mizoram | 11.8 |  |  |
| Takhar | 16.5 | Nagaland | 23.2 |  |  |
| Urozgan | 0.0 | Orissa | 9.3 |  |  |
| Wardak | 9.5 | Pondicherry | 22.5 |  |  |
| Zabul | 0.0 | Punjab | 18.2 |  |  |
| **BANGLADESH, 2018** |  | Rajasthan | 20.2 |  |  |
| Barisal | 13.6 | Sikkim | 16.7 |  |  |
| Chittago | 20.4 | Tamil Nadu | 17.1 |  |  |
| Dhaka | 14.5 | Telangana | 15.9 |  |  |
| All numbers are percentages | | | | | |
