# Supplementary material for: More is not enough: High quantity and high quality antenatal care are both needed to prevent low birthweight in South Asia
Source: PLOS Glob Public Health. 2023 Jun 8;3(6):e0001991. doi: 10.1371/journal.pgph.0001991 (PMC10249805; doi:10.1371/journal.pgph.0001991)
Supplement: S3 Table — (DOCX) [file pgph.0001991.s004.docx]

|  | Afghanistan 2015 | Bangladesh 2018 | India  2016 | Nepal  2016 | Pakistan  2018 | Sri Lanka^1^  2016 |
| --- | --- | --- | --- | --- | --- | --- |
|  | n=2,454 | n=2,179 | n=131,732 | n=2,574 | n=1.187 | n=6,158 |
|  | *OR*  *95% CI* | *OR*  *95% CI* | *OR*  *95% CI* | *OR*  *95% CI* | *OR*  *95% CI* | *OR*  *95% CI* |
| Combination of ANC quantity and quality (ref: Low quantity and low quality) | | | | | | |
| Low quantity and high quality | 0.53  0.27:1.05 | 0.84  0.50:1.42 | 0.90**  0.84:0.96 | 1.10  0.65:1.85 | 0.49  0.23:1.05 | 1.17  0.91:1.51 |
| High quantity and low quality | 0.77  0.45:1.29 | 1.05  0.50:2.20 | 1.06  0.94:1.20 | 1.10  0.56:2.14 | 1.08  0.42:2.79 | 0.75**  0.61:0.93 |
| High quantity and high quality | 1.22  0.63:2.33 | 0.78  0.48:1.29 | 0.84***  0.78:0.89 | 0.58*  0.35:0.94 | 0.45*  0.23:0.86 | 0.73**  0.57:0.92 |
| Women's age at survey, years | 1.00  0.95:1.05 | 0.99  0.96:1.03 | 1.00  0.99:1.00 | 0.96*  0.92:1.00 | 0.98  0.94:1.03 | 1.00  0.99:1.02 |
| Women’s education (r*ef: no education)* | | | | | | |
| Primary | 0.44**  0.25:0.80 | 0.55  0.29:1.07 | 1.01  0.94:1.09 | 0.74  0.48:1.15 | 1.00  0.5:1.99 | 0.80  0.39:1.65 |
| Secondary | 0.47*  0.23:0.96 | 0.39**  0.21:0.74 | 0.90***  0.85:0.96 | 0.71  0.47:1.07 | 1.67  0.85:3.27 | 0.58  0.30:1.12 |
| Higher | 0.17*  0.04:0.67 | 0.37**  0.19:0.73 | 0.69***  0.63:0.76 | 0.84  0.52:1.38 | 0.67  0.31:1.44 | 0.44*  0.22:0.87 |
| Women's BMI<18.5 kg/m^2^ | **-** | 0.88  0.59:1.32 | 1.33***  1.27:1.40 | 1.39  0.92:2.10 | 0.89  0.32:2.49 | 1.4**  1.12:1.74 |
| First child | 1.58  0.87:2.88 | 1.16  0.78:1.71 | 1.17***  1.11:1.24 | 1.33  0.94:1.90 | 1.03  0.59:1.82 | 1.29*  1.04:1.59 |
| Female child | 2.13***  1.51:3.00 | 1.33*  1.03:1.72 | 1.22***  1.17:1.28 | 1.42*  1.08:1.86 | 0.96  0.65:1.44 | 1.42***  1.21:1.65 |
| Household is rural | 0.36***  0.21:0.62 | 0.95  0.70:1.29 | 0.97  0.91:1.03 | 1.08  0.80:1.47 | 0.93  0.57:1.51 | 1.04  0.85:1.28 |
| Household wealth quintile (*ref: Poorest)* | | | | | | |
| Second | 0.52  0.21:1.29 | 0.95  0.57:1.59 | 0.94  0.88:1.00 | 1.27  0.72:2.22 | 1.18  0.44:3.18 | 0.72**  0.56:0.92 |
| Third | 1.02  0.50:2.06 | 0.92  0.54:1.57 | 0.90**  0.84:0.97 | 1.07  0.64:1.80 | 0.97  0.37:2.59 | 0.69**  0.53:0.89 |
| Fourth | 0.86  0.31:2.42 | 0.76  0.45:1.28 | 0.88**  0.82:0.96 | 1.45  0.87:2.42 | 0.77  0.27:2.17 | 0.53***  0.39:0.72 |
| Richest | 0.63  0.29:1.35 | 0.72  0.40:1.29 | 0.78***  0.71:0.86 | 0.98  0.55:1.76 | 0.78  0.27:2.24 | 0.60**  0.42:0.88 |
| Women’s height and weight was not measured in Afghanistan DHS, as a result we were not able to calculate the BMI. Logistic regression adjusted for states or divisions fixed effects.  ^1^In Sri Lanka, high ANC quality was defined as receiving 10 out of 10 interventions and high ANC quantity was defined as at least 8 ANC visits due to limited variation in ANC quantity and quality. ***p<0.001, **p<0.01 *p<0.05. | | | | | | |
