# Supplementary material for: More is not enough: High quantity and high quality antenatal care are both needed to prevent low birthweight in South Asia
Source: PLOS Glob Public Health. 2023 Jun 8;3(6):e0001991. doi: 10.1371/journal.pgph.0001991 (PMC10249805; doi:10.1371/journal.pgph.0001991)
Supplement: S4 Table — (DOCX) [file pgph.0001991.s005.docx]

|  | Afghanistan  2015 | | Bangladesh  2018 | | India  2016 | | Nepal  2016 | | Pakistan  2018 | |
| --- | --- | --- | --- | --- | --- | --- | --- | --- | --- | --- |
|  | With BW data | Without BW data | With BW data | Without BW data | With BW data | Without BW data | With BW data | Without BW data | With BW data | Without BW data |
| N | 2,783 | 16,906 | 2,279 | 2,733 | 1,47,674 | 43,035 | 2,618 | 1,388 | 1,621 | 6,665 |
| Women’s characteristics |  |  |  |  |  |  |  |  |  |  |
| Age at survey, years | 28.7 | 28.9 | 24.8 | 25.0* | 27.0 | 28.5*** | 25.8 | 27.4*** | 29.9 | 29.5 |
| Education |  |  |  |  |  |  |  |  |  |  |
| No education*, %* | 69.4 | 87.6*** | 2.8 | 9.1*** | 22.0 | 52.3*** | 20.9 | 49.4*** | 16.4 | 58.7*** |
| Primary*, %* | 11.9 | 6.3*** | 15.4 | 38.1*** | 13.2 | 16.8*** | 17.1 | 22.8*** | 11.7 | 13.7 |
| Secondary*, %* | 14.0 | 5.0*** | 50.9 | 45.5*** | 51.9 | 28.4*** | 40.6 | 23.9*** | 31.9 | 18.5*** |
| Higher*, %* | 4.6 | 1.0*** | 31.0 | 7.3*** | 12.9 | 2.5*** | 21.4 | 4.0*** | 40.0 | 9.2*** |
| BMI<18.5 kg/m^2^, % | - | - | 12.7 | 19.6*** | 22.7 | 27.4*** | 14.4 | 22.4*** | 4.8 | 10.2*** |
| Number of births |  |  |  |  |  |  |  |  |  |  |
| 1, % | 19.8 | 14.1*** | 47.6 | 30.4*** | 36.5 | 18.4*** | 46.6 | 20.5*** | 25.8 | 17.5*** |
| 2, % | 17.1 | 15.4 | 32.6 | 32.8 | 34.7 | 26.0*** | 30.1 | 28.7 | 26.4 | 19.9*** |
| ≥3, % | 63.0 | 70.5*** | 19.9 | 36.8*** | 28.8 | 55.6*** | 23.3 | 50.9*** | 47.8 | 62.6*** |
| Children's characteristics |  |  |  |  |  |  |  |  |  |  |
| Female, % | 45.1 | 48.2 | 46.2 | 48.8 | 45.5 | 46.7** | 43.8 | 46.3 | 48.1 | 48.0* |
| Age, months | 20.8 | 21.0* | 16.6 | 16.4 | 25.0 | 26.2*** | 24.9 | 27.7* | 21.7 | 22.8 |
| Household's characteristics |  |  |  |  |  |  |  |  |  |  |
| Rural, % | 60.7 | 78.5*** | 56.4 | 73.3*** | 71.6 | 86.3*** | 33.6 | 56.8*** | 38.4 | 58.9*** |
| Wealth Quintile |  |  |  |  |  |  |  |  |  |  |
| Poorest, % | 8.4 | 21.9*** | 9.7 | 28.7*** | 15.0 | 37.3*** | 13.1 | 33.0*** | 3.5 | 24.0*** |
| Second, % | 13.0 | 21.2*** | 15.0 | 24.4*** | 18.0 | 26.7*** | 16.5 | 26.5*** | 9.1 | 22.6*** |
| Third, % | 17.1 | 20.4*** | 17.8 | 21.4* | 20.4 | 18.5*** | 19.9 | 20.1 | 16.3 | 20.9*** |
| Fourth, % | 24.2 | 19.3 | 24.5 | 17.2*** | 22.4 | 11.6*** | 23.5 | 13.5*** | 26.8 | 18.4*** |
| Richest, % | 37.4 | 17.1*** | 33.0 | 8.2*** | 24.1 | 5.9*** | 26.9 | 6.9*** | 44.4 | 14.1*** |
| ***p<0.001, **p<0.01 *p<0.05  Note: Because there were only 130 children without birthweight in Sri Lanka, we did not compare samples with and without birthweight.  BW, birthweight | | | | | | | | | | |
