# Supplementary material for: More is not enough: High quantity and high quality antenatal care are both needed to prevent low birthweight in South Asia
Source: PLOS Glob Public Health. 2023 Jun 8;3(6):e0001991. doi: 10.1371/journal.pgph.0001991 (PMC10249805; doi:10.1371/journal.pgph.0001991)
Supplement: S5 Table — (DOCX) [file pgph.0001991.s006.docx]

|  | Afghanistan  2015 | India  2016 | | Nepal  2016 | | Pakistan  2018 | |  |
| --- | --- | --- | --- | --- | --- | --- | --- | --- |
|  | n=17,021 | n=165,503 | n=3,942 | | n=6,630 | |  |  |
|  | *OR*  *95% CI* | *OR*  *95% CI* | *OR*  *95% CI* | | *OR*  *95% CI* | |  |  |
| Combination of ANC quantity and quality (r*ef:* Low quantity and low quality) | | | | | | |  |  |
| Low quantity and high quality | 1.00  0.77:1.31 | 0.82***  0.76:0.87 | 0.80  0.56:1.13 | | 0.95  0.74:1.23 | |  |  |
| High quantity and low quality | 0.95  0.74:1.23 | 0.92  0.82:1.03 | 0.69  0.43:1.11 | | 1.07  0.78:1.45 | |  |  |
| High quantity and high quality | 0.96  0.74:1.26 | 0.80***  0.74:0.85 | 0.62**  0.45:0.84 | | 0.89  0.71:1.12 | |  |  |
| Woman’s age at survey, years | 1.01  1.00:1.02 | 0.99***  0.99:1.00 | 1.00  0.98:1.02 | | 0.99  0.97:1.00 | |  |  |
| Women’s education (r*ef: no education)* | | | | | | |  |  |
| Primary | 1.23  0.97:1.55 | 1.01  0.94:1.07 | 1.05  0.79:1.40 | | 0.86  0.65:1.13 | |  |  |
| Secondary | 0.95  0.77:1.18 | 0.92**  0.87:0.98 | 0.88  0.63:1.22 | | 0.79  0.60:1.05 | |  |  |
| Higher | 0.38**  0.21:0.70 | 0.82***  0.74:0.91 | 0.96  0.66:1.39 | | 0.71  0.50:1.01 | |  |  |
| Women's BMI<18.5 kg/m^2^ | **-** | 1.23***  1.17:1.29 | 1.28  0.97:1.69 | | 1.16  0.85:1.58 | |  |  |
| First child | 1.24*  1.02:1.50 | 1.10***  1.04:1.16 | 1.32*  1.01:1.72 | | 0.98  0.76:1.26 | |  |  |
|  |  |  |  | |  | |  |  |
| Child is female | 1.33***  1.18:1.50 | 1.17***  1.12:1.22 | 1.34**  1.12:1.60 | | 1.09  0.92:1.28 | |  |  |
|  |  |  |  | |  | |  |  |
| Household is rural | 0.76*  0.61:0.95 | 1.03  0.95:1.10 | 0.99  0.79:1.24 | | 1.03  0.81:1.30 | |  |  |
| Household wealth quintile *(ref: poorest)* | | | | | | |  |  |
| Second | 1.04  0.88:1.23 | 0.93*  0.87:0.99 | 1.06  0.79:1.43 | | 1.17  0.91:1.49 | |  |  |
| Third | 0.90  0.73:1.12 | 0.91*  0.85:0.98 | 0.87  0.65:1.17 | | 1.02  0.77:1.34 | |  |  |
| Fourth | 0.94  0.74:1.19 | 0.91*  0.84:0.98 | 0.91  0.66:1.25 | | 0.83  0.60:1.15 | |  |  |
| Richest | 0.78  0.60:1.02 | 0.78***  0.71:0.86 | 0.74  0.52:1.07 | | 0.60**  0.42:0.88 | |  |  |
| ***p<0.001, **p<0.01 *p<0.05. Women’s height and weight was not measured in Afghanistan DHS, as a result we were not able to calculate BMI in Afghanistan. Logistic regression was adjusted for states or divisions fixed effect. | | | | | | | | |
