# Supplementary material for: More is not enough: High quantity and high quality antenatal care are both needed to prevent low birthweight in South Asia
Source: PLOS Glob Public Health. 2023 Jun 8;3(6):e0001991. doi: 10.1371/journal.pgph.0001991 (PMC10249805; doi:10.1371/journal.pgph.0001991)
Supplement: S6 Table — recalled birthweight and combination of ANC quantity and quality, by country in South Asia. (DOCX) [file pgph.0001991.s007.docx]

|  | Afghanistan  2015 | | India  2016 | | | Nepal  2016 | | | Pakistan  2018 | |
| --- | --- | --- | --- | --- | --- | --- | --- | --- | --- | --- |
|  | Recorded | Recalled | Recorded | Recalled | Recorded | | Recalled | Recorded | | Recalled |
|  | n=1,088 | n=1,278 | n=72,363 | n=59,369 | n=514 | | n=2,060 | n=197 | | n=990 |
|  | *OR*  *95% CI* | *OR*  *95% CI* | *OR*  *95% CI* | *OR*  *95% CI* | *OR*  *95% CI* | | *OR*  *95% CI* | *OR*  *95% CI* | | *OR*  *95% CI* |
| Combination of ANC quantity and quality (r*ef:* Low quantity and low quality) | | | | | | | | | | |
| Low quantity and high quality | 0.69  0.29:1.62 | 0.50  0.21:1.21 | 0.86**  0.78:0.95 | 0.94  0.86:1.02 | 5.11  0.73:35.93 | | 0.84  0.49:1.45 | 0.13**  0.03:0.53 | | 0.59  0.25:1.38 |
| High quantity and low quality | 0.85  0.38:1.89 | 0.73  0.3:1.76 | 1.16  0.94:1.42 | 0.99  0.85:1.15 | 4.54  0.57:35.92 | | 0.98  0.48:1.99 | 0.34  0.04:3.16 | | 1.25  0.41:3.77 |
| High quantity and high quality | 0.89  0.35:2.24 | 1.77  0.76:4.13 | 0.79***  0.72:0.87 | 0.91*  0.83:0.99 | 0.79  0.12:5.4 | | 0.61*  0.37:0.99 | 0.04***  0.01:0.19 | | 0.61  0.29:1.29 |
| Woman’s age at survey, years | 1.04  0.97:1.11 | 0.97  0.91:1.02 | 1.01  1.00:1.01 | 0.99**  0.98:1.00 | 0.97  0.9:1.04 | | 0.96  0.93:1.00 | 1.02  0.92:1.13 | | 0.98  0.94:1.03 |
| Women’s education (r*ef: No education)* | | | | | | | | | | |
| Primary | 0.28*  0.10:0.79 | 0.69  0.23:2.07 | 1.41***  1.31:1.51 | 1.24***  1.16:1.33 | 0.36  0.09:1.51 | | 0.85  0.54:1.33 | 1.67  0.41:6.78 | | 0.89  0.41:1.94 |
| Secondary | 0.52  0.18:1.47 | 0.49*  0.24:0.99 | 0.98  0.88:1.09 | 1.05  0.96:1.15 | 1.04  0.33:3.29 | | 0.69  0.47:1.01 | 1.09  0.21:5.61 | | 1.68  0.81:3.48 |
| Higher | 0.17  0.02:1.31 | 0.15**  0.05:0.50 | 0.91*  0.84:0.99 | 0.89**  0.82:0.96 | 1.19  0.33:4.31 | | 0.78  0.47:1.3 | 1.70  0.24:11.91 | | 0.60  0.27:1.35 |
| Women's BMI<18.5 kg/m^2^ | **-** | **-** | 0.72***  0.63:0.82 | 0.66***  0.58:0.75 | 0.76  0.24:2.36 | | 1.51  0.98:2.35 | 1.92  0.08:45.64 | | 0.82  0.28:2.45 |
| First child | 2.00  0.84:4.77 | 1.18  0.53:2.60 | 1.25***  1.16:1.34 | 1.08  1.00:1.16 | 1.90  0.87:4.17 | | 1.28  0.87:1.9 | 1.39  0.34:5.75 | | 1.04  0.57:1.9 |
| Child is female | 2.90**  1.55:5.45 | 1.54  0.91:2.61 | 1.23***  1.16:1.31 | 1.21***  1.15:1.28 | 1.06  0.55:2.04 | | 1.51**  1.12:2.03 | 2.77  0.64:11.97 | | 0.89  0.57:1.37 |
| Household is rural | 0.40*  0.20:0.80 | 0.37*  0.17:0.81 | 0.94  0.87:1.02 | 1.01  0.93:1.10 | 0.59  0.3:1.16 | | 1.19  0.85:1.66 | 1.4  0.46:4.26 | | 0.98  0.6:1.58 |
| Household wealth quintile *(ref: Poorest)* | | | | | | | | | | |
| Second | 0.31*  0.11:0.88 | 0.74  0.21:2.65 | 0.96  0.87:1.05 | 0.92  0.83:1.01 | 0.56  0.12:2.59 | | 1.36  0.75:2.46 | 0.65  0.06:6.57 | | 1.24  0.42:3.65 |
| Third | 0.92  0.34:2.46 | 1.14  0.4:3.27 | 0.91  0.82:1.00 | 0.90*  0.81:0.99 | 1.07  0.33:3.49 | | 0.89  0.52:1.53 | 0.95  0.1:9.17 | | 0.97  0.35:2.66 |
| Fourth | 0.84  0.32:2.16 | 0.87  0.2:3.82 | 0.90  0.81:1.01 | 0.86**  0.77:0.96 | 0.77  0.24:2.45 | | 1.44  0.86:2.41 | 1.66  0.15:18.71 | | 0.72  0.25:2.07 |
| Richest | 0.47  0.15:1.4 | 0.86  0.3:2.42 | 0.80***  0.70:0.90 | 0.76***  0.66:0.86 | 0.69  0.18:2.6 | | 0.95  0.51:1.76 | 1.26  0.08:19.08 | | 0.77  0.27:2.18 |
| ***p<0.001, **p<0.01 *p<0.05. Women’s height and weight was not measured in Afghanistan DHS, as a result we were not able to calculate BMI in Afghanistan. Logistic regression was adjusted for states or divisions fixed effect. | | | | | | | | | | |
