# Supplementary material for: More is not enough: High quantity and high quality antenatal care are both needed to prevent low birthweight in South Asia
Source: PLOS Glob Public Health. 2023 Jun 8;3(6):e0001991. doi: 10.1371/journal.pgph.0001991 (PMC10249805; doi:10.1371/journal.pgph.0001991)
Supplement: S7 Table — (DOCX) [file pgph.0001991.s008.docx]

|  | Afghanistan n=17,028 | Bangladesh n=2,179 | India n=165,676 | Nepal n=1,872 | Pakistan  n=2,132 | Sri Lanka n=6,158 |
| --- | --- | --- | --- | --- | --- | --- |
|  | *OR*  *95% CI* | *OR*  *95% CI* | *OR*  *95% CI* | *OR*  *95% CI* | *OR*  *95% CI* | *OR*  *95% CI* |
| Combination of ANC quantity and quality (ref: Low quantity and low quality) | | | | | | |
| Low quantity and high quality | 0.81  0.60:1.10 | 0.84  0.50:1.42 | 0.94*  0.89:0.99 | 0.79  0.48:1.3 | 0.77  0.50:1.19 | 1.17  0.91:1.51 |
| High quantity and low quality | 0.84  0.67:1.06 | 1.05  0.50:2.20 | 1.06  0.96:1.18 | 0.94  0.49:1.81 | 0.93  0.56:1.53 | 0.75**  0.61:0.93 |
| High quantity and high quality | 1.03  0.83:1.28 | 0.78  0.48:1.29 | 0.88***  0.83:0.93 | 0.53**  0.35:0.83 | 0.66*  0.45:0.96 | 0.73**  0.57:0.92 |
| Women's age at survey, years | 1.01  1.00:1.02 | 0.99  0.96:1.03 | 1.00  0.99:1.00 | 1.00  0.96:1.04 | 1.00  0.97:1.02 | 1.00  0.99:1.02 |
| Women’s education ref: No education | | | | | | |
| Primary | 1.07  0.82:1.40 | 0.55  0.29:1.07 | 1.05  0.98:1.11 | 0.79  0.51:1.22 | 1.16  0.74:1.82 | 0.80  0.39:1.65 |
| Secondary | 0.83  0.65:1.06 | 0.39**  0.21:0.74 | 0.96  0.91:1.01 | 0.78  0.48:1.28 | 1.14  0.72:1.81 | 0.58  0.30:1.12 |
| Higher | 0.36**  0.19:0.68 | 0.37**  0.19:0.73 | 0.74***  0.68:0.81 | 1.14  0.62:2.08 | 0.92  0.55:1.55 | 0.44*  0.22:0.87 |
| Women's BMI<18.5 kg/m^2^ | - | 0.88  0.59:1.32 | 1.31***  1.26:1.37 | 2.00***  1.40:2.86 | 1.56*  1.02:2.36 | 1.4**  1.12:1.74 |
| First child | 1.3*  1.06:1.60 | 1.16  0.78:1.71 | 1.17***  1.12:1.23 | 1.34  0.85:2.11 | 1.14  0.75:1.75 | 1.29*  1.04:1.59 |
| Child is female | 1.35***  1.2:1.53 | 1.33*  1.03:1.72 | 1.19***  1.15:1.23 | 1.50*  1.10:2.05 | 1.29  0.97:1.73 | 1.42***  1.21:1.65 |
| Household is rural | 0.74**  0.59:0.92 | 0.95  0.70:1.29 | 0.97  0.92:1.02 | 1.10  0.81:1.48 | 0.82  0.56:1.22 | 1.04  0.85:1.28 |
| Household wealth quintile ref: Poorest | | | | | | |
| Second | 1.02  0.86:1.21 | 0.95  0.57:1.59 | 0.96  0.90:1.01 | 1.04  0.63:1.73 | 1.13  0.69:1.85 | 0.72**  0.56:0.92 |
| Third | 0.9  0.73:1.12 | 0.92  0.54:1.57 | 0.93*  0.87:0.99 | 0.81  0.46:1.41 | 0.69  0.42:1.15 | 0.69**  0.53:0.89 |
| Fourth | 0.86  0.64:1.14 | 0.76  0.45:1.28 | 0.91**  0.85:0.97 | 0.74  0.44:1.25 | 0.96  0.54:1.73 | 0.53***  0.39:0.72 |
| Richest | 0.74*  0.57:0.97 | 0.72  0.40:1.29 | 0.82***  0.76:0.89 | 0.74  0.41:1.34 | 0.68  0.38:1.23 | 0.60**  0.42:0.88 |
| ***p<0.001, **p<0.01 *p<0.05. Women’s height and weight was not measured in Afghanistan DHS, as a result we were not able to calculate BMI in Afghanistan. Logistic regression was adjusted for states or divisions fixed effect. In Bangladesh and Sri Lanka due to missing data on perceived size, results are from primary analysis | | | | | | |
