# Supplementary material for: More is not enough: High quantity and high quality antenatal care are both needed to prevent low birthweight in South Asia
Source: PLOS Glob Public Health. 2023 Jun 8;3(6):e0001991. doi: 10.1371/journal.pgph.0001991 (PMC10249805; doi:10.1371/journal.pgph.0001991)
Supplement: S8 Table — (DOCX) [file pgph.0001991.s009.docx]

|  |
| --- |

|  | AF 2015 | | BD 2018 | | IN 2016 | | NP 2016 | | PK 2018 | | LK 2016 | |
| --- | --- | --- | --- | --- | --- | --- | --- | --- | --- | --- | --- | --- |
|  | % | n | % | n | % | n | % | n | % | n | % | n |
| Moderate low birthweight (≥2000 & <2500 gm) | 11.8 | 342 | 10.5 | 240 | 13.3 | 19,058 | 8.0 | 215 | 14.1 | 204 | 12.2 | 848 |
| Very low birthweight (≥1500<2000 gm) | 1.8 | 51 | 3.5 | 72 | 2.9 | 4,250 | 2.5 | 71 | 4.2 | 58 | 2.1 | 140 |
| Extremely low birthweight (<1500 gm) | 2.2 | 30 | 1.4 | 30 | 1.2 | 1722 | 0.9 | 21 | 4.5 | 49 | 1.0 | 66 |
| AF=Afghanistan; BD=Bangladesh; IN=India; NP=Nepal; PK=Pakistan; LK= Sri Lanka. | | | | | | | | | | | | |
