# Supplementary material for: More is not enough: High quantity and high quality antenatal care are both needed to prevent low birthweight in South Asia
Source: PLOS Glob Public Health. 2023 Jun 8;3(6):e0001991. doi: 10.1371/journal.pgph.0001991 (PMC10249805; doi:10.1371/journal.pgph.0001991)
Supplement: S9 Table — (DOCX) [file pgph.0001991.s010.docx]

|  | India  (n=131,732) | |
| --- | --- | --- |
|  | Moderately LBW (≥2000 & <2500 gm) | Very/extremely LBW (<2000 gm) |
|  | *RRR 95% CI* | *RRR 95% CI* |
| Combination of ANC quantity and quality (ref: Low quantity and low quality) |  |  |
| Low quantity and high quality | 0.91**(0.84:0.97) | 0.87*(0.77:0.99) |
| High quantity and low quality | 0.97(0.84:1.12) | 1.33*(1.07:1.65) |
| High quantity and high quality | 0.86***(0.80:0.92) | 0.77***(0.67:0.89) |
| Women's age at survey, years | 1.21***(1.15:1.27) | 1.27***(1.17:1.38) |
| Women’s education ref: No education | 1.12***(1.05:1.18) | 1.38***(1.25:1.52) |
| Primary | 1.00(0.99:1.00) | 1.01(1.00:1.02) |
| Secondary | 1.32***(1.25:1.4) | 1.37***(1.26:1.49) |
| Higher |  |  |
| Women's BMI<18.5 kg/m^2^ | 1.00(0.93:1.09) | 1.04(0.92:1.18) |
| First child | 0.89***(0.83:0.95) | 0.94(0.85:1.05) |
| Child is female | 0.68***(0.61:0.75) | 0.74**(0.62:0.89) |
| Household is rural | 0.98(0.92:1.05) | 0.92(0.83:1.02) |
| Household wealth quintile ref: Poorest |  |  |
| Second | 0.91*(0.84:0.98) | 1.03(0.91:1.17) |
| Third | 0.90*(0.83:0.98) | 0.90(0.79:1.02) |
| Fourth | 0.88**(0.81:0.96) | 0.90(0.78:1.04) |
| Richest | 0.78***(0.70:0.86) | 0.80*(0.67:0.95) |
| ***p<0.001, **p<0.01 *p<0.05. *RRR=Relative Risk Ratio. Multinominal logistic* regression was adjusted for states or divisions fixed effect. In Bangladesh and Sri Lanka due to missing data on perceived size, results are from primary analysis | | |
