# Supplementary material for: More is not enough: High quantity and high quality antenatal care are both needed to prevent low birthweight in South Asia
Source: PLOS Glob Public Health. 2023 Jun 8;3(6):e0001991. doi: 10.1371/journal.pgph.0001991 (PMC10249805; doi:10.1371/journal.pgph.0001991)
Supplement: Attachment — Submitted filename: Response to reviewers.docx [file pgph.0001991.s017.docx]

**Journal Requirements:**

1. Please amend your detailed Financial Disclosure statement. This is published with the article. It must therefore be completed in full sentences and contain the exact wording you wish to be published.
a. State what role the funders took in the study. If the funders had no role in your study, please state: “The funders had no role in study design, data collection and analysis, decision to publish, or preparation of the manuscript.”
b. If any authors received a salary from any of your funders, please state which authors and which funders.

Author response: We have added “The funders had no role in study design, data collection and analysis, decision to publish, or preparation of the manuscript.” to the funding statement.

2. Please provide separate figure files in .tif or .eps format.

For more information about figure files please see our guidelines:  LINK

<https://journals.plos.org/globalpublichealth/s/figures> 
<https://journals.plos.org/globalpublichealth/s/figures#loc-file-requirements>

We have provided separate figure files in .tif format.

3. We have noticed that you have uploaded Supporting Information files, but you have not included a list of legends. Please add a full list of legends for your Supporting Information files after the references list.

Author response: We have added a list of legends for supplemental files after the references list in the manuscript document.

4. In the online submission form, you indicated that "We used the Demographic and Health Survey data, which is available from DHS website upon request.". All PLOS journals now require all data underlying the findings described in their manuscript to be freely available to other researchers, either 1. In a public repository, 2. Within the manuscript itself, or 3. Uploaded as supplementary information.
   This policy applies to all data except where public deposition would breach compliance with the protocol approved by your research ethics board. If your data cannot be made publicly available for ethical or legal reasons (e.g., public availability would compromise patient privacy), please explain your reasons by return email and your exemption request will be escalated to the editor for approval. Your exemption request will be handled independently and will not hold up the peer review process, but will need to be resolved should your manuscript be accepted for publication. One of the Editorial team will then be in touch if there are any issues.

Author response: We have uploaded data underlying the findings as supplementary information.

Additional Editor Comments (if provided):

This is overall interesting paper that covers an area of great interest. Factors that are related to prevent low birthweight in South Asia. The authors have made a very good effort overall to describe their findings, considering this is a topic highly quantitative. There are however areas they further need to address.

Were data analyzed clustering at country level? This can highly affect results.

Author response: A separate model was run for each country.

**Comments to the Author**

5. Review Comments to the Author

Please use the space provided to explain your answers to the questions above. You may also include additional comments for the author, including concerns about dual publication, research ethics, or publication ethics. (Please upload your review as an attachment if it exceeds 20,000 characters)

Reviewer #1: The descriptive statistics sub-section could be more informative. Inference could be deduced from differences in the sample characteristics. Comparing country statistics and exploiting the variance could be a meaningful addition.

Author response: We appreciate the reviewer’s suggestion. A separate regression model was run for each country (we did not pool data and model the association between ANC quantity/quality and LBW overall in the region), hence we do not focus on comparing country statistics such as differences in education level or number of births. These factors are included as predictors in each model as they may confound the association between ANC quantity/quality and LBW.

Further, comparing the size of the parameter estimates between countries and providing plausible reasons while the odds in Nepal, for instance, is different from India, and whether quality and quantity could be explanation.

Author response: While the point estimates for India and Nepal differ, the confidence intervals overlap so, statistically speaking, the estimates do not differ from each other. As the reviewer rightly points out, the magnitude of the point estimate does differ, however, and suggests that the combination of high quantity and high quality ANC appears to be more protective against LBW in Nepal (OR=0.57, p<0.05) vs. India (OR=0.84, p>0.001). We would like to point out that the interventions including in the ANC quality indicator differ by country (e.g. in Nepal, data on weight assessment, ultrasound, food supplementation and health/nutrition education were not available whereas in India they were). These differences make it difficult to compare estimates across countries, and this is also part of why we did not pool data and conduct a regional analysis.

Reviewer #2: This manuscript uses nationally representative household surveys using Demographic and Household data from six South Asian countries to examine the relationship between quantity and/or quality of womens ANC visit and LBW infants. This is an important body of research given the attention on vulnerable newborns and the research offers important insights for policy.

In general, it would be helpful to provide further comparisons of the differences across the South Asian countries and why these differences may exist in terms of prevalence of LBW infants and/or differences in QoC received by mothers. One smaller addition that would be helpful and benefit the readership would benefit this analysis is to provide a breakdown of LBW infants into moderately LBW, very LBW and Extremely LBW infants to see the prevalence, burden and note if ANC is related to any one of these in particular given the larger sample size.

Author response: We thank the reviewer for his/her comments. We have conducted an additional analysis based on the suggestion where we break down birth weight into categories of extremely LBW (ELBW; <1500 grams), very LBW (VLBW; 1500-1999 grams) and moderate LBW (MLBW; 2000-2499 grams) following the definitions of these categories per the World Health Organization (International statistical classification of diseases and related health problems, 10^th^ revision, 2^nd^ ed. World Health Organization; 2004.). The sample sizes for these categories are a constraint in all countries except India. For India, we ran additional multinomial logistic regression models predicting MLBW and VLBW or ELBW (we combined the latter two as the sample sizes for each were too small to run individual models). As expected, the protective effect of high quantity and high quality ANC (compared to low quantity and low quality ANC) on VLBW or ELBW (<2000 grams) is slightly stronger than it is for LBW (<2500 grams) (OR=0.77 for VLBW/ELBW vs. 0.84 for LBW), though the confidence intervals overlap. We have added this additional analysis in the supplemental information (Supplemental Tables 8 and 9) and mentioned it in the Results text under sub-section Robustness checks. In terms of differences across countries, it is challenging to draw specific conclusions given the different policies, programs and interventions in each country, and we feel that the available data preclude this type of comparison (which is why we also chose not to pool data and conduct a regional analysis with all countries combined).
